# Dr. Samuel Brown as an Author

**Published:** 1854-09

**Authors:** L. P. Yandall


### Art. II.—DR. SAMUEL BROWN AS AN AUTHOR.

EXTRACT FROM AN UNPUBLISHED REPORT ON THE MEDICAL LITERATURE OF KENTUCKY, BY L. P. YANDELL, M. D.

A movement was made about the close of the last century towards the organization of the Medical Department of Transylvania University. Dr. Samuel Brown was elected Professor of the Theory and Practice of Medicine and Chemistry in 1799, and about the same time Dr. Frederick Ridgely, who had distinguished himself as a surgeon in the revolutionary army, delivered a course of lectures to a small class in the University. The latter, so far as we have been able to learn, has left no fruits of his pen behind him. He was a pupil and correspondent of Dr. Rush, and a physician of learning and skill. Dr. Brown has the credit, we believe, of having produced the first medical paper in Kentucky to be found on record. After graduating in Edinburgh, then the most famous school of medicine in the world, he settled in Lexington and shortly afterwards was honored with the appointment mentioned, but gave no lectures under it. About this time he became a contributor to the New York *Medical Repository*, at that time the only journal of medicine published in the United States. His communications were occasional and brief, consisting of familiar letters addressed to one of the editors. His first is not the one which bears the earliest date; it is dated Lexington, June, 1799, and is to be found in the fourth volume of the Medical Repository, p. 225, and is entitled "*A Curious Instance af Disease in which the feeling of the patient was abolished, while the power of motion remained unimpaired.*" The papers of Dr. Brown have an intrinsic interest, and besides this there is a curiosity about them as the earliest specimens of our medical literature. For this reason we shall give a somewhat copious abstract of his communications.

The subject of the first case was a lady of Bardstown, aged about forty years, and when Dr. Brown saw her, had been deprived for more than two years of sensation in her hands and feet. She was quite insensible to the effects of cutting instruments, or of having coals applied to them. "In one instance," Dr. B. remarks, "when she was engaged in shaping a piece of wood with a knife, she incautiously turned her eyes upon some other object, and cut off the end of the thumb of her left hand, without perceiving the smallest sense of pain. She cannot," continued Dr. B., "from her sensations, discover the least difference between a hot and a cold iron, and has frequently burnt the skin and flesh to a considerable depth by mistaking the one for the other. These wounds and burns heal without any uncommon difficulty. Notwithstanding this total loss of sensibility, she retains the power of motion in full perfection, and pursues her domestic employments without any remarkable inconveniece. All her animal and vital functions are in a natural, healthful state, and her spirits are regular,—nay, even cheerful. She feels no uneasiness from her complaint, except a sense of fulness in the veins. As the sense of touch is entirely lost, she finds it difficult to retain substances in her hands, and on turning her eyes aside often drops glasses, plates, etc., which she holds in safety as long as she looks at them."

In concluding his report Dr. Brown remarks: "The doctrine of the nervous system is yet so much embarrassed with controverted facts and visionary theories that I shall not attempt an explanation of the phenomena of the above case. It is only by carefully noting and recording facts that we can hope to arrive at truth in our physiological investigations." He lived to see much of the obscurity which rested upon this system cleared up, and light and order substituted for visionary theories.

In the same volume of the *Reposttory* on a previous page (p. 223,) we have the report of a case by the same writer, dated November 10, 1800. It is entitled "Extraordinary case of a Man who appeared to have *Three Testicles*," The subject was a married man, thirty-seven years of age, the father of several children, and in excellent health. About two months before applying to Dr. Brown, he perceived a slight uneasiness in the ring of the abdominal muscle on the right side, and felt a small tumor, which was easily pushed back into the abdomen. It was suspended by a distinct cord, which, "in size and feeling exactly resembled those of the other two testicles." Dr. Brown felt distinctly the action of the cremaster muscle as often as he directed the patient to retract the testicles, and was fully persuaded, as were also Dr. E. Warfield and Dr. Marshall who examined the case at his request, that the tumor was a veritable testicle.

This volume of the *Repository* contains the history of another remarkable case by Dr. Brown. A young lady, aged about 19 years, punctured her foot with a nail, which gave rise to the most acute pain in the limb, extending to the knee, the hip, and the back. Tetanic rigidity followed. "A physician was sent for who unluckily was at a horse race and declined to visit her. He, however, sent a few anodyne pills which she took, but without any material advantage." The accident occurred on Sunday the 28th of October, and Dr. Brown saw her the Wednesday following. He found the patient in extreme agony, with spasms in her back and neck, and acute pains in her ancle, knee, and hip joint. Not the slightest appearance of inflammation could be discerned in the puncture, but the heel was cold, the skin pale, and nothing but blood and serum could be pressed from the wound. He introduced a probe and was surprised to find that she was scarcely sensible of pain from a very harsh examination of the extent and direction of the puncture. At length the point of his probe came in contact with a part which possessed extreme sensibility. He made an incision with a scalpel across the heel quite down to the bone, on which the whole of the complaints seemed to be removed, and he flattered himself that he should be able to prevent a recurrence of the spasms. He washed the wound with spirits of turpentine, and having made a dossil of lint he covered it with powdered sulphate of copper and spirits of turpentine and pressed it down to the bottom of the incision. This application gave severe pain. A large dose of laudanum was given and repeated every four hours; a blister was also applied to the ancle, and a warm poultice to the heel. Two grains of calomel were ordered every hour when the patient was awake, and an ounce of mercurial ointment was rubbed into the legs, arms and back; and Dr. Brown adds, that he endeavored to hasten salivation by rubbing calomel on the gums. She slept about two hours, and continued tolerable easy until the next day, when her spasms returned and all the symptoms were greatly aggravated. Wine and bark, which had proved so efficacious in the hands of Dr. Rush, were tried, but the stomach immediately rejected them. Two ounces of mercurial ointment had now been rubbed in, twenty-two grains of calomel had been taken in the form of pills, and a scruple rubbed on the gums, when Dr. Brown, despairing of being able to excite ptyalism in time to prevent a fatal termination of the disease, directed fifteen drops of the tincture of cantharides every hour, until the symptoms of inflammation in the bowels should forbid its further exhibition. Hot poultices were continued to the wound, which was filled with the root of the phytolacca decandra. I conclude the history of the case in the words of the author.

"After the patient had taken ℥jss. of the tincture of cantharides, she was seized with a sensation of heat and burning in the stomach, which, becoming more and more severe, extended through the whole course of the alimentary canal, and produced a number of stools, mixed with blood and mucus. The inflammation of the stomach occasioned vomiting, and the acute pains in her bowels resembled those which are felt in the most violent attacks of dysentery. *Immediately on the occurrence of these symptoms, all the tetanic affections disappeared, and never returned.* In order to mitigate the disease which I had produced in the alimentary canal I gave large quantities of mucilaginous drinks, and anodyne enemata, which afforded considerable ease. Fortunately, however, the day after this inflammatory state of the bowels came on, the mercury, which before seemed quite inert, began to show its effect on the salivary glands, and (as we have often seen in dysentery), removed the disease of the intestines. Some slight affection of the kidneys appeared, with bloody urine, but soon left the patient without any complaint, except a very moderate salivation, which continued for about two weeks. The wound began to form pus eight days after the accident, and was kept open several weeks, from an apprehension, that if closed, the spasms might return."

Dr. Brown closes his history of this interesting case with the following remarks:

"This case, which terminated so happily, is not communicated to the public as the foundation of any new favorite theory. The writer is sufficiently aware of the danger of deducing general inferences from solitary facts. Disappointed in the expectation of relieving his patient by the fashionable remedies, his practice was the result of that necessity which occasionally gives birth to the most useful discoveries. He thought it his duty to communicate it with simplicity and candor, and hopes that those who practice medicine in climates where tetanus is a common disease, will strive to lessen its mortality, by giving cantharides a fair trial. It is well known that physicians are often under the necessity of combating one disease by creating another, and although it must be confessed that the inflammation of the stomach and bowels is attended with great danger, no practitioner will hesitate to affirm that tetanus is still more dangerous."

The fifth volume of the same work contains the details of an interesting case of *poisoning*, in a child, by the seeds of the *Datura stramonium*, communicated by Dr. Brown in a letter to Dr. Mitchill, one of the editors of the *Repository*. "On the 5th of April, 1801, he says, I was called a few miles from Lexington, to visit a child of Mr. George Bryan, aged two years. The following history of her complaint was given me by her relations. About 10 o'clock of the same day, she was observed to catch at the blaze of the fire in a very singular manner, and a few minutes after fall on the floor as if she had been paralysed on one side. In a short time symptoms of violent fever came on, with delirium and a scarlet efflorescence over the whole body, which had not entirely disappeared when I saw her first, at 12 o'clock, P. M. This efflorescence was much more bright and lively than that which characterizes scarlatina. Through the day the delirium continued with short intervals of reason. An unceasing disposition for motion in all the muscles, weeping, screaming, laughter, and rapid incoherent exclamation, formed the leading features of this disease, which to me was both new and alarming. Once or twice the child had made feeble efforts to vomit, which induced the family to believe that worms were the cause of these extraordinary symptoms. But I was impressed, from the beginning, with a firm conviction, that there was something in the case which I had not before seen in my practice. On presenting a candle to the child, I discovered an unusual dilatation of the pupils, and a remarkable squinting of the right eye, which, however, was not constant. The pulse was extremely irregular; sometimes it was hurried and fluttering, sometimes it was tense and strong, and frequently its action was not discernible. The motions of the limbs and body resembled those which mark the highest grade of

*chorea sancti viti*, but were much more convulsive and violent, and excited in the spectators rather a *painful* than a *ridiculous* emotion. Contemplating the case with great solicitude, it occurred to me that these strange symptoms might originate from the effects of the datura stramonium on the stomach, and I immediately inquired if that plant grew near the place, but was answered in the negative. On repeating my inquiries, however, one of the children informed me that she had given some seeds to another sister who was not indisposed, but firmly asserted tnat none had been taken by the one who was ill. This discovery, although not completely satisfactory, rendered my first conjecture still more probable, and I determined to act agreeably to it. I therefore administered a strong dose of emetic tartar. It was long before I could produce nausea; and as the first efforts to which were abortive, I made use of a feather, which excited one feeble motion. No seeds were discharged; and I found great difficulty, even by the feather, in awakening the powers of the stomach. I had lately read some remarks of M. Humboldt, on the possibility of renovating the excitability by alkalies, after exhaustion by opium."

Carbonate of potash was accordingly given, "and had not reached the stomach two minutes," he continues, "when full vomiting took place, and seven seeds of the stramonium were thrown into a plate which Mr. Bryan held that he might have an opportunity of proving the event of my prognostic." The little patient slept well afterwards, and though there were some convulsive symptoms with wildness of the countenance, next morning, in a day or two every complaint was removed."

In vol. II, p. 145, of the same work, Dr. B. gives an account of "*Two Cases of Convulsions, alternating with Insanity, relieved by violent pressure over the stomach.*" The cases were extraordinary. In the first, a man about twenty-five years of age was seized with convulsions while excited in an argument with a friend. The convulsive movements were succeeded by insanity, which, after a few moments, gave way to convulsions again. Before Dr. B. despaired of the efficacy of antispasmodics, he had given his patient two ounces of laudanum without producing any sensible effect. Recollecting, then, that he had seen cases of hysteria relieved by a bandage tied tightly round the body, he attempted to apply one in this case, and in doing so pressed with some force against the stomach. He perceived an immediate change in the countenance of the patient, and on increasing the pressure the young man smiled and expressed himself relieved. On withdrawing his hand all the symptoms recurred, and were again relieved by the pressure. "From twelve o'clock until four," continues Dr. B., "the experiment was repeated at least ten times, with uniformly the same result. When the convulsions were strong, my own force was not sufficient to check them. Another person's strength was added occasionally. At four o'clock the patient went to sleep with my *whole weight* resting on my knee, which was placed over his stomach!" He gradually withdrew, and next morning found his patient free from spasms, with his understanding composed.

The second patient was a lady, about twenty years of age, who had been subject from her infancy to convulsions of an hysterical character, in one of which Dr. Brown found her. He says, "I immediately pressed my knuckles with great force into the pit of the stomach, and in less than five minutes she was free from convulsions, and her mind composed. I withdrew my hand, and the complaint returned, as in the other case. I left a person with her to continue the pressure, and found her well the next day."

Dr. Brown was a man of discursive mind and did not by any means limit his studies to medicine. He made short contributions to the American journals of science, none of them elaborate, but generally mere notices of some striking or curious phenomenon. In *Bruce's Journal* (vol. I., p. 100,) he has a brief account of the "Nitre caves of Kentucky;" and the sixth volume of the *Transactions of the American Philosophical Society* contains a paper from him on the same subject. He wrote to Prof. Silliman, July, 1818, describing "a curious substance which accompanies the native Nitre of Kentucky and of Africa," and his letter is contained in the first volume of the *American Journal of Science and Art.*

This, we believe, is the sum of Dr. Brown's contributions to the literature of his profession. He was not an author, although he possessed all the learning and the talents necessary to have rendered him a successful one. He had enjoyed the advantages of a thorough classical and professional education, and his natural gifts were of a high order. His powers of observation were admirable; he was quick in his perceptions; wrote with ease, fluency, and grace, and in conversation was singularly pleasing, witty, brilliant, and impressive. His style of lecturing was conversational, and his lectures, delivered sitting, abounded in anecdote and humor. But he was averse to severe labor. He did not long pursue any inquiry, or adhere to any system, but rather amused himself with the novelties of the profession, which his active, inquisitive mind was sure to be one of the first to seize. It is to this cause that we are to ascribe the failure of a man so gifted by nature and so highly favored by fortune, to attain a high rank among American medical writers. He was Professor of the Theory and Practice of Medicine in Transylvania University from 1819 to 1825.

Dr. Brown was the founder of the "*Kappa Lambda Society of Hippocrates*," an institution framed by his benevolent mind to bring harmony to the profession. He hoped to see every worthy physician in America enrolled among the members of the society, and thus, by becoming brethren in a double sense, be made to exhibit a fraternal spirit in their intercourse with each other. The conception fired his mind with all the zeal of an enthusiast, and he pursued the object with a full assurance of success. A journal of medicine, "*The North American Journal of Medicine and Surgery*," was set on foot in Philadelphia, in 1826, under the auspices of this society, and at once took a high rank for learning and ability among the medical journals of the country. It was edited by Drs. Bell, Coates, Hodge, Meigs, and La Roche, personal friends of Dr. Brown and members of the society. Dr. Brown died in Alabama, the 12th of January, 1830.

